# Engineering Crystal Packing in RNA-Protein Complexes II: A Historical Perspective from the Structural Studies of the Spliceosome

**DOI:** 10.3390/cryst11080948

**Published:** 2021-08-15

**Authors:** Adelaine Kwun-Wai Leung, Yasushi Kondo, Daniel A. Pomeranz Krummel, Jade Li, Stephen R. Price, Anne-Marie M. van Roon

**Affiliations:** 1 Department of Veterinary Biomedical Sciences, University of Saskatchewan, Saskatoon, SK S7N 5B4, Canada; 2 Department of Molecular and Cell Biology, University of California, Berkeley, CA 94720, USA; 3 California Institute for Quantitative Biosciences, University of California, Berkeley, CA 94720, USA; 4 Department of Neurology and Rehabilitation Medicine, University of Cincinnati College of Medicine, Cincinnati, OH 45267, USA; 5 Structural Studies Division, MRC Laboratory of Molecular Biology, Cambridge CB2 0QH, UK; 6 Research Department of Cell and Developmental Biology, UCL Division of Biosciences, London WC1E 6DE, UK

**Keywords:** crystallization, RNA-protein complexes, spliceosome

## Abstract

Cryo-electron microscopy has greatly advanced our understanding of how the spliceosome cycles through different conformational states to conduct the chemical reactions that remove introns from pre-mRNA transcripts. The Cryo-EM structures were built upon decades of crystallographic studies of various spliceosomal RNA-protein complexes. In this review we give an overview of the crystal structures solved in the Nagai group, utilizing many of the strategies to design crystal packing as described in the accompanying paper.

## 1 Introduction

**Figure 1:**
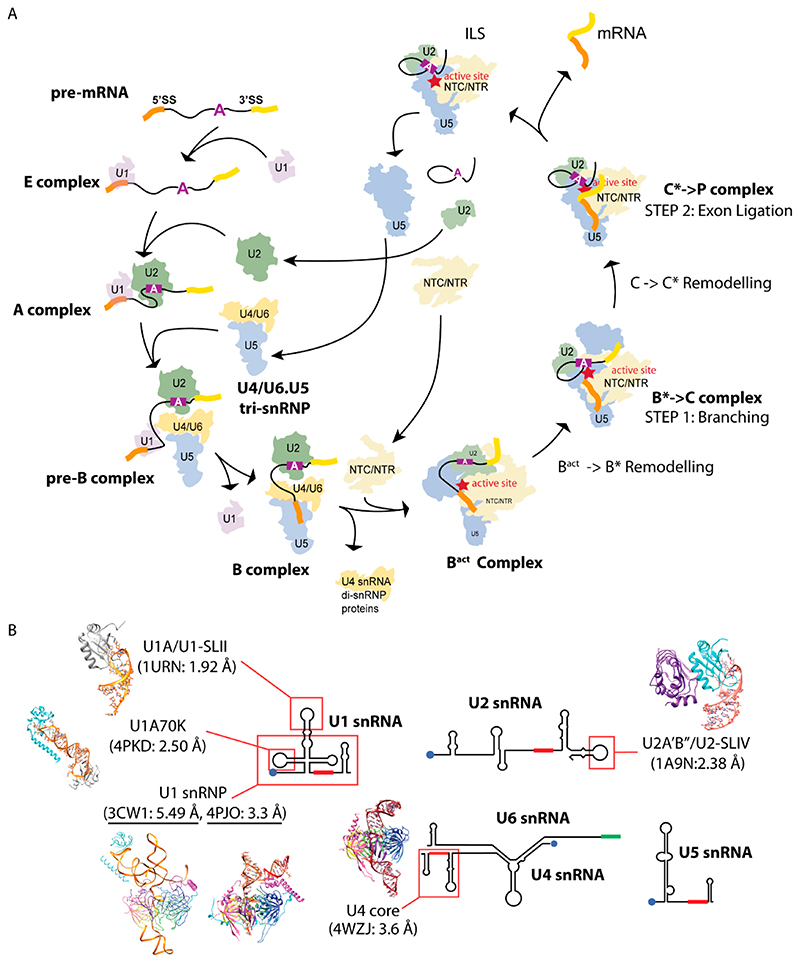
(**A**) The spliceosome in different catalytic states as revealed by Cryo-EM. Shown are the schematics of the pre-mRNA and the silhouettes of each splicing complex from Cryo-EM structures. Proteins involved in remodeling the spliceosome are not included. NTC/NTR are protein complexes that help sculpt the active site of the spliceosome together with U2, U5, and U6 snRNPs. *Prespliceosome (E, A complex)*: U1 snRNP recognizes the 5′ splice site (5′ss) forming the E complex. U2 snRNP recognizes and the invariant adenosine on the branch point (purple box) forming the A complex. *Precatalytic spliceosome (Pre-B, B Complex)*: The U4/U6.U5 tri-snRNP enters to form the Pre-B complex and the helicase-dependent dissociation of U1 snRNP generates the B complex. Activated spliceosome (B^act-^Complex, B^*^, C, C^*^): Further helicase-dependent remodeling releases U4 snRNA and U4/U6 di-snRNP proteins, which allow U6 snRNA to refold with U2 snRNA and the pre-mRNA into a catalytic active conformation, allowing the 2-step splicing reactions to occur. In step 1, the 2*′*-OH of the branch point adenosine attacks the phosphorous of the 5′ss to form the lariat intron. In step 2, the 3′-OH of the cleaved 5′ss attacks the phosphorous of the 3′ss to form the ligated mRNA. *Postspliceosomal complex (P complex)*: Helicase-dependent release of the ligated exons. *Disassembly of the spliceosome*: Helicase-dependent release of intron lariat and recycling of U snRNPs. (**B**) Secondary structures of the U snRNAs and a summary of crystal structures of spliceosomal RNA-protein complexes determined by the Nagai lab from 1994 to 2015. The U snRNPs share seven common Sm proteins that assemble on the Sm site (in red solid box). Blue dots represent the tri-methylguanosine cap. U6 snRNA does not have an Sm site but instead contains a U-rich tail that is bound by seven paralogs of the Sm proteins (LSm 2–8). *U1A/U1-SLII*: the hairpin loop II of U1 snRNA bound to the U1A protein was determined to be 1.92 Å in 1994. *U2′AB″/U2-SLIV*: the hairpin loop IV of U2 snRNA bound to the U2 specific proteins U2A*′* and U2B*″* was determined to be 2.38 Å in 1998. *U4 core*: the core domain structure of the U4 snRNP containing two stem loops flanking the Sm site and seven Sm proteins was determined in stages to the final refined structure at 3.6 Å (2005, 2011, 2016). *U1 snRNP*: The first U1 snRNP structure was determined without U1A and the hairpin loop II to be 5.5 Å in 2009. The minimal snRNP, with the four-way junction replaced by a stem loop and the N-terminal U1-70K peptide fused to SmD1, which was determined to be 3.3 Å in 2015. *U1A70K*: the hairpin loop I of U1 snRNA bound to U1-70K was determined to be 2.50 Å in 2015.

The spliceosome is a dynamic macromolecular “machine” responsible for removing introns and splicing together exons from eukaryotic precursor-mRNA transcripts (pre-mRNAs). The spliceosome comprises five large RNA-protein complexes or Uridine-rich small nuclear ribonucleoprotein particles (U snRNPs). Each U snRNP is named after its snRNA component (U1, U2, U4, U5, and U6 snRNAs), and collectively they contain approximately 170 proteins. Other than the seven Sm proteins that are commonly found in U1, U2, U4, and U5 snRNPs forming the core domain, most proteins are specific to each U snRNP. These U snRNPs assemble onto specific regions of the pre-mRNA and participate through different states of the splicing cycle [1] (Figure 1A). The E complex forms when U1 snRNP binds to the 5′ exon-intron boundary or splice site (5′ss). The binding of U2 snRNP at an invariant adenosine within the intron establishes the pre-spliceosome A complex. The recruitment of the U4/U6-U5 tri-snRNP forms the pre-B spliceosome. Afterward, the spliceosome undergoes dynamic remodeling via multiple conformational changes to allow for catalysis (two trans-esterification reactions) to occur (See review Wilkinson, 2020). Since the “resolution revolution” of electron cryo-microscopy or Cryo-EM [2,3], high-resolution structures of the spliceosome in different states of the splicing cycle have been reported (http://spliceosomedb.ucsc.edu/structures, accessed on 11 August 2021). These structures have dramatically increased our understanding of the molecular mechanism of RNA splicing (see reviews [4–6]). Crystal structures of spliceosomal components solved previously were fit into the Cryo-EM maps, thereby greatly facilitating model building (Figure 1B). Here, we review the Nagai group’s effort in solving X-ray structures of RNA-protein spliceosomal complexes utilizing crystal packing design strategies discussed in the accompanying paper [7].

## 2 Determining Spliceosomal Protein-Hairpin Structures

The hairpin loop II bound to protein U1A (U1A/U1-SLII) from the U1 snRNP and hairpin loop IV bound to the two proteins U2A′/B*″* (U2A′B″/U2-SLIV) from the U2 snRNP were the first structures of spliceosomal RNA-protein complexes the Nagai group elucidated [8]. One challenge for hairpin loops is that the RNA has a high affinity to form a self-dimer, instead of folding into a hairpin structure that can be bound by the protein. Therefore, optimizing an annealing protocol to ensure proper folding of the RNA and an assay that can confirm binding of the protein is the first requirement before engineering crystal packing [9]. In these projects, the rationale for RNA engineering involved optimizing end-to-end packing of the stem by creating overhangs and optimizing its length and sequence. For both structures, extensive protein engineering was performed in combination with different RNA constructs [9–11].

**Figure 2:**
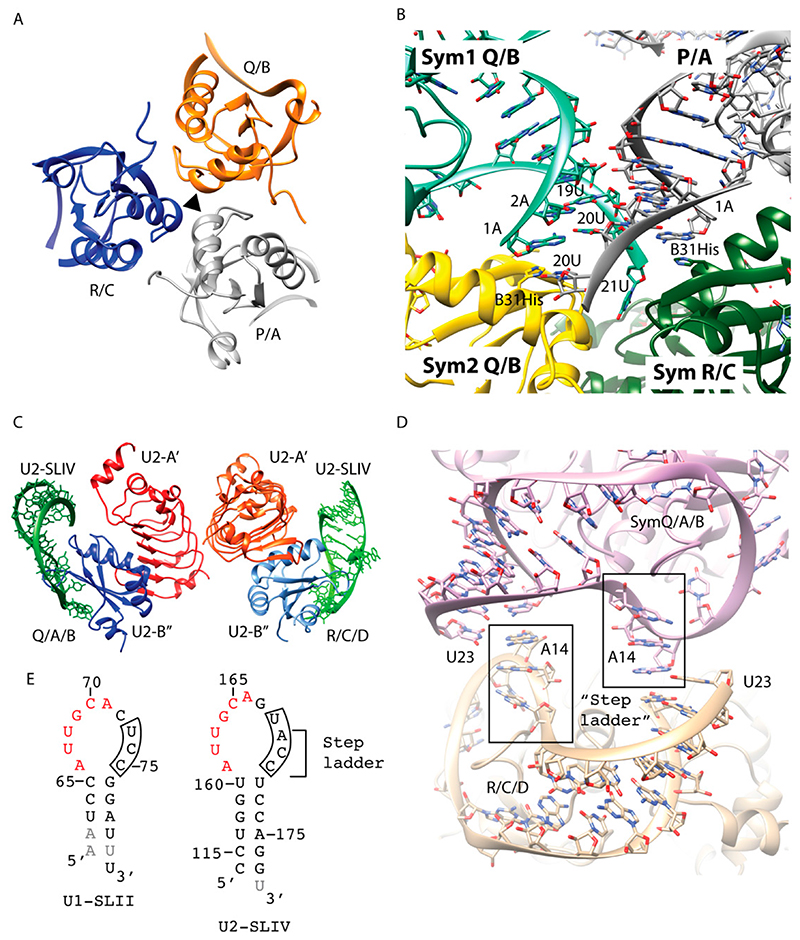
Hairpin structures of U1-SLII and U2-SLIV. (*A*-*B*) *U1A/U1-SLII (PDB:1URN)*. (**A**) Three NCS copies related by a 3-fold axis are present in the asymmetric unit. The complex is named after the chain ID. A, B, C: U1A and P, Q, R: U1-SLII. Only protein-protein interactions are observed between the NCS complexes. (**B**) Close up of the protein-RNA interaction between the NCS copies. The Y31H splits open the end pair (A1:U20) of the stem loop of P/A. (*C*-*D*) *U2A′B″/U1-SLIV (PDB:1A9N)*. (**C**) Two NCS copies are present in the asymmetric unit. The complex is named after the chain ID. A, C:U2-A′; B, D:U2-B″; Q, R:U2-IV. Interactions between U2-A′ are the only contacts between the NCS complexes. (**D**) The sequence A14, C15, C16 at the 3′ end of the loop forms a step ladder structure. A14 forms a Hoogsteen to WC base pair with the 3′ sticky U23. (**E**) The hairpin loop sequence of U1-SLII and U2-SLIV. The 5′ 6 nt loop sequence (in red) are identical between the two RNAs whereas the 3′ loop sequence confers binding specificity. Engineered sequence is colored in gray.

For the U1A/U1-SLII crystal structure, the construct combination that yielded diffracting crystals were a 21-nt RNA composed of the hairpin loop with a single U overhang at the 3′ end (Figure 2E) and an U1A construct with two surface mutations [10]. One of the U1A mutations was engineered to disrupt a crystal contact that predominated a poorly diffracting crystal form, and the other (Y31H) was engineered serendipitously through a PCR error [10]. In the final 1.92 Å crystal structure, the three complexes (P/A, Q/B, and R/C; named after their chain IDs), related by an NCS 3-fold, were present in the asymmetric unit [12] (Figure 2A). Only protein-protein interactions were involved at the NCS interface. The end-to-end RNA packing did not occur as designed. Nevertheless, the ends of the duplex did make critical crystal contacts. The RNA-RNA contacts made by each NCS complex and its symmetry-related partners were slightly different. In general, the backbones of the RNA stems made a series of ribose-zipper-like interactions, in which the 2′OH from one RNA duplex hydrogen bonded with the sugar edge base from another duplex (Figure 2B). The sticky 3′ U21 was only fully ordered in one NCS copy and Watson-Crick (WC) base pairing of the last bp (1A:20U) was not consistently present. The accidental mutation Y31H made several key crystal packing interactions. The P/A complex interacted with three symmetry-related molecules of the Q/B and R/C complexes (Figure 2B). In the P/A complex, instead of base pairing between A1 and U20, each nucleotide interacted separately with Y31H from the two symmetry-related complexes, causing this pair to split up. U20 from the P/A complex hydrogen bonded with ND1 of Y31H from one symmetry-related Q/B complex (Sym2), which, in turn, stacked with the 1A:20U end pair of another symmetry-related Q/B complex (Sym1). On the other strand, the unpaired A1 was stabilized by Y31H from the symmetry-related R/C complex, making stacking interaction to the base (Figure 2B).

U2B” is homologous to U1A, and its binding to the stem loop U2-SLIV requires U2A′. The 6-nt (AUUGCA) at the 5′ end of the U2-SLIV hairpin loop is identical to that of U1-SLII (Figure 2E). Utilizing similar strategies to optimize end-to-end packing of the RNA stems, the final RNA construct used to crystallize U2-SLIV/U2A’B” complex had 24 nts and a 3′ U overhang to create a sticky end. The final 2.38 Å crystal structure contained two ternary complexes (Q/A/B and R/C/D, named after their chain IDs) interacting with each other via the U2-A’ protein in the asymmetric unit (Figure 2C). Similar to the case for the U1-SLII/U1A structure, end-to-end duplex packing did not occur. However, the 3′ sticky end nucleotide U23 did make key crystal packing contacts interacting with the hairpin loop sequence that confers binding specificity discriminating between U1A and U2A′/B″ (Figure 2D). The crystal structure of U1-SLII/U1A showed that the last 3 nts of the loop sequence (UCC) did not contact the protein and in two NCS copies, these nucleotides were disordered (Figure 2E). In contrast, the 3′ loop nucleotides of U2-SLIV/U2A′B″ (UACC) made extensive interactions with the protein and nucleotides A14, C15, C16 formed a step ladder facing the solvent (Figure 2D,E). The Watson edge of the 3′ sticky U23 formed a WC to Hoogsteen bp with A14 (Figure 2D).

In summary, these early spliceosomal hairpin structures foretold the reality of engineering crystal contacts, in that the packing may not occur as designed but the engineered element still interacted with specific structural motifs available in each complex. Most importantly, these projects led to the development of an in vitro transcription system that allowed us to create large quantities of RNA with homogeneous ends, setting the stage for more complicated RNA engineering for the next series of larger RNA-protein complexes [13].

## 3 Utilizing the Tetraloop and Tetraloop Receptor RNA Motif to Crystallize the U4 snRNP Core Domain

**Figure 3:**
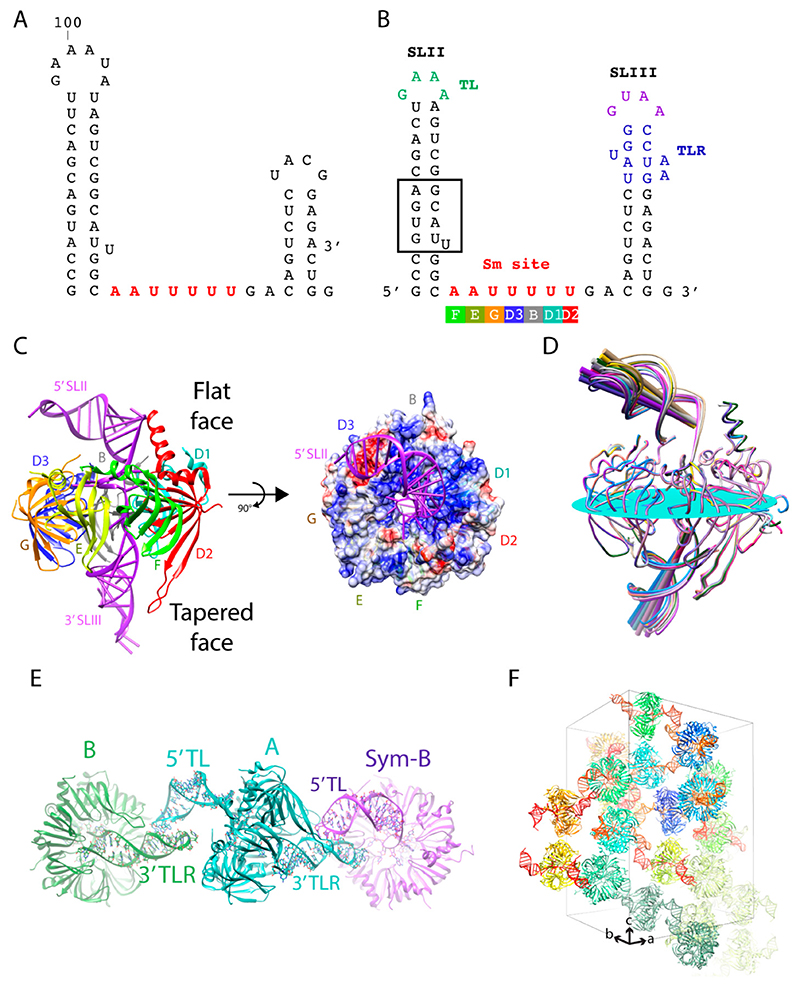
(**A**) Native sequence of the 3′ end of the U4 snRNA. (**B**) U4 construct used in the crystal structure. Highlighted in green is the GAAA tetraloop (TL), in purple is another stable GRNA tetraloop, and in blue is the GAAA receptor (TLR). The Sm site nucleotides that interact around the inner pore of the core ring is highlighted in red. Each Sm site nucleotide interacts in the binding pocket of the Sm protein depicted underneath. (**C**) One complex of the U4 snRNP core domain. The 5′ SLII carrying the GAAA tetraloop is located on the flat face of the core ring and the 3′ SLIII carrying the TLR is located on the tapered face of the core ring. The 5′ SLII bends over the D2/D1/B sector of the ring. The surface color represents the electric potential of the surface (blue: positive, red: negative). (**D**) Superposition of the 12 NCS copies showing the stem loops have variable tilt angles relative to the plane of the core ring. The plane and the axes were drawn using Chimera. The plane was drawn by selecting the first residue after helix 1 of each Sm-fold. The orientation of the superposition is adjusted slightly from that shown in Figure A to better highlight the tilt angles of the RNA stems. (**E**) One example of the TL/TLR interactions between symmetry pairs. Twelve complexes packed as six distinct pairs are in the asymmetric unit. On the flat face, the 5′ TL of complex A interacts with the 3′ TLR of the NCS complex B. On the tapered face, the 3′ TLR of complex A interacts with another complex B related by crystallographic symmetry. (**F**) The TL/TLR interactions combines the core rings to pack rim-to-rim along the c axis.

The core domain is a common structural scaffold present in U1, U2, U4, and U5 snRNPs. The RNA components of these snRNPs share a conserved single-stranded region called the Sm site upon which the seven Sm proteins (D1, D2, D3, B, E, F, and G) are assembled (Figure 3). To visualize the architecture of this recurrent structural domain, the quest to crystallize the core domain began. Prior to this work, crystal structures of two sub-complexes of the core domain, the D1D2 and D3B heterodimers, revealed a common fold and protein interface between the Sm proteins [14]. By incorporating these building principles with biochemical data, a ring model comprising the seven Sm proteins was proposed [14]. How the heptameric ring recognizes the Sm site specifically and the location of the flanking RNAs were unknown. The U4 snRNP core domain was selected because the Sm site of the U4 snRNA is immediately flanked by two stem loops, whereas other U snRNAs have longer single-stranded regions that may induce flexibility undesirable for crystallization (Figure 1B). With the in vitro transcription system that allowed us to efficiently prepare any RNA sequence for crystallization in place, we first generated the truncated U4 snRNA (SLII + Sm site + SLIII) with native sequence (Figure 3A). No crystals were obtained with core domain complex reconstituted with this RNA or with a construct where the stems were shortened and capped with GNRA tetraloops (TL) [15]. Next, we designed a series of constructs with engineered crystal packing motifs at different positions on the stem. The native sequence was maintained for the bottom 6–7 bp of the stem as we rationalized that the region close to the Sm site may make critical interactions with the core ring. We obtained several different crystal forms with constructs containing a tetraloop and its tetraloop receptor (TLR) on each stem to promote a “head-to-tail” interaction as described previously [15]. The best crystal diffracted anisotropically to 3.4 Å along c^*^ but only 4 Å normal to it. The final crystal structure of the U4 snRNP core domain contained seven Sm proteins bound to the truncated U4 snRNA with the tetraloop on the 5′ SLII and its receptor engineered on the 3′ SLIII (Figure 3B). The tetraloop and its receptor interacted as designed, bringing together the core rings to stack rim-to-rim in a column along the c-axis (Figure 3F). Interactions between the core rings involved mostly van der Waals contacts. Therefore, the long-range interactions between the inserted TL/TLR motifs were responsible for contacts in directions perpendicular to the c-axis to establish the three-dimensional lattice. The engineered contacts were strong and allowed the crystals to diffract to high resolution. The crystal belonged to the space group *P*3_1_ with 12 complexes in the asymmetric unit. The complexes were packed as six distinct pairs via the engineered crystal contacts. The 5′ TL from one complex (A) interacted with the 3′ TLR of an NCS-related complex (B) and the 3′ TLR of complex A interacted with the 5′ TL of the crystallographic symmetry-related complex B (Sym-B) (Figure 3E).

**Figure 4:**
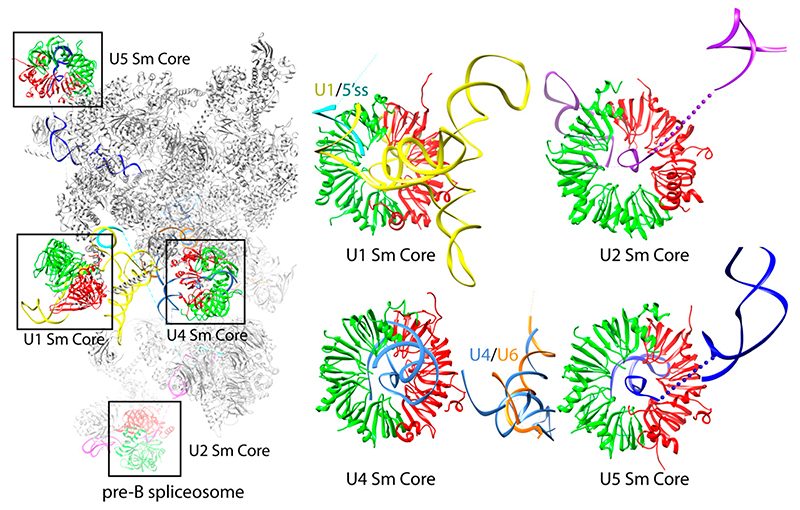
The 3.3 Å Cryo-EM structure of Pre-B spliceosome captured before U1 snRNP dissociates (PDB:6QX9). The model is depicted in ribbons representation. The D2/D1/B sector of the four core domains is colored in red and D3/G/E/F is colored in bright green; all other protein components are colored in grey. U1 snRNA (yellow), U2 snRNA (purple), U4 snRNA (light blue), U5 snRNA (dark blue), U6 (orange), and pre-mRNA (cyan). The functionally important RNA structures all bend toward the D2/D1/B sector. Thus, the direction of the RNA backbone 5′ to the Sm site is significant in positioning structural elements that eventually form the catalytic active site.

The placement of the TL and its TLR allowed the stem loops to interact consistently, but also with room to accommodate variations in the tilt angle relative to the plane of the core ring. The angle of the 5′ SLII varied from 21.9° to 34.7° (the RNA construct used in theΔ12.8°), whereas the 3′ stem varied from 46.4° to 54.1° (Δ7.7°) (Figure 3D). The tilt angle variation allowed for an optimal protein-protein interaction between the heptameric rings packed in columns along crystal’s c-axis (Figure 3F). However, the combination of the TL/TLR being the only interactions coupled to the variable tilt angle between the stem loops could have contributed to the weaker anisotropic diffraction in the ab plane and the tetartohedral twinning that made structure determination challenging [16,17]. Based on the packing of this crystal form, further attempts to engineer the RNA by introducing more motifs to stabilize the 5′ stem continued. We tried inserting the paromomycin binding site containing two flip-out adenines to promote potential lateral contacts of the 5′ SLII and adding a 5′ single strand extension carrying a triple G motif that had the potential to form a quadruplex crystal contact [7]. Although new crystal forms were obtained with these new constructs, they did not improve diffraction [15]. Nonetheless, the U4 core domain structure refined against a 3.6 Å data set provided important biological insights into the core domain. It revealed the mechanism of Sm site recognition and how the RNA threaded through the central hole with the 5′ SLII and 3′ SLIII located on the flat face and tapered face of the core ring, respectively [16,17] (Figure 3C). Upon exiting out of the core ring, the 5′ SLII bends over to the D2/D1/B sector on the flat face of the core ring (Figure 3C). Although the D2/D1/B sector has a more electropositive potential (colored blue in Figure 3C), electrostatic interaction cannot be responsible for the bending because of the large vertical distance between the backbone of the 5′ SLII stem and the core ring (Figure 3D). Therefore, the direction of bending is constrained by non-canonical base pairing in the ring-proximal segment, which includes the GU wobble pair, the single U asymmetric internal loop, and the AG pair (Figure 3B boxed). In subsequent structures of larger spliceosomal complexes, the bending of the 5′ stem toward the D2/D1/B sector is maintained in the U4 snRNP with native sequences [18,19]. The NCS copy with the most bent 5′ stem is the only complex that fitted the U4/U6.U5 tri-snRNP Cryo-EM map [18]. Although U1, U2, and U5 snRNAs have variable RNA structures 5′ to the Sm site compared to U4 snRNA, their backbones also curve toward the D2/D1/B sector of their respective core domains (Figure 4) [18–23]. The curvature could be governed by their own RNA structural elements and stabilized by interaction with the N- and C-terminal extensions of D2/D1/B located on the flat face of the core ring. The extensions can be adapted to provide additional contacts to accommodate different RNA structures, particularly the functionally important RNA elements located 5′ to the Sm site in all U snRNAs (Figure 1A). The pliability of these N- and C-terminal extensions is demonstrated in the core domains of U4 and U1 snRNPs. In the U4 core domain structure, the N-terminal extension (H0) of D2 was ordered into a helix in several NCS copies while others remained disordered. Whether or not H0 made contact with the RNA seemed to be dependent on the degree of RNA bending [16,17]. In the U1 snRNP structure, which has an intact 4-way junction 5′ to the Sm site, the H0 of D2 appeared as an ordered long helix to contact the RNA [24]. The coordinates of the U4 snRNP core domain (4WZJ) have been used as the model template to build the core domains of U2, U4, and U5 snRNPs in all subsequent Cryo-EM structures [18–22].

## 4 Utilizing the Kissing Loop Motif to Crystalize U1 snRNP

**Figure 5:**
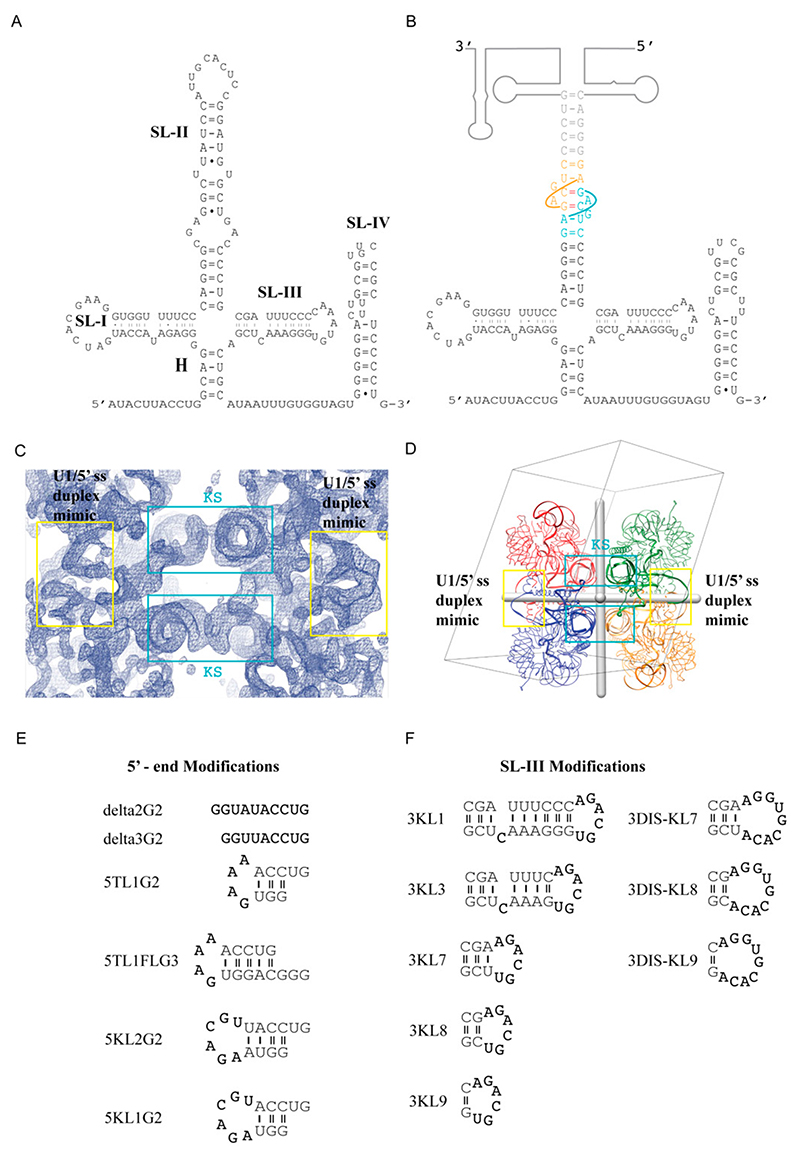
(**A**) Native sequence of U1 snRNA. (**B**) Construct used to crystallize the first U1 snRNP. The base pairing between the kissing loop is depicted in cyan and orange. (**C**) Experimental electron density map of U1 snRNP at 5.5 Å. Density for the major and minor grooves of the kissing loop helices and the U1/5′ss helices is clear. (**D**) The packing arrangement of U1 snRNP for the *P*1 crystal form (PDB:3CW1). The four NCS complexes are related by three orthogonal 2-fold symmetry axes (shown as gray rods). The kissing loop interactions are formed along one 2-fold. The 5′ strand of the U1 snRNA base pair with its NCS-related partner along another 2-fold axis, mimicking the U1/5′ss interaction. (**E**,**F**) Summary of the next series of constructs modified to improve diffraction quality of the *P*1 crystal form. Examples of constructs with modified 5′ end (**E**) and modified SL-III (**F**).

The U1 snRNP recognizes the 5′ splice site of a pre-mRNA to form the spliceosomal E complex via base-pairing with the 5′ end of U1 snRNA. In addition to the seven Sm proteins of the core domain, human U1 snRNP (~240 kDa) has three additional proteins: U170K, U1A, and U1C. Flanking the single-stranded Sm site of the U1 snRNA is one stem loop (SL-IV) on the 3′ side and four stem loops (SL-I-III and H) connected by a four-way junction, which co-axial stack, on the 5′ side (Figure 5A).

In a biochemical tour-de-force, all ten proteins of human U1 snRNP were produced by heterologous expression in bacteria and reconstituted with an in vitro transcribed U1 snRNA. This complex was purified and shown to be functional [25,26]. Using native gel electrophoresis and mass spectrometry, we further characterized this fully recombinant complex and showed it be compositionally homogeneous [25–27]. This set the stage for crystallization; however, the fully recombinant particle did not yield crystals when a significant number of particle variants were generated. It was previously shown that the protein U1A was dispensable for U1 snRNP activity [28,29] and therefore, a variant of the recombinant particle was reconstituted lacking U1A. In addition, a ‘kissing loop’ motif was introduced in U1 snRNA in place of the U1A binding site on U1 snRNA. Specifically, a U1 snRNA variant used for crystallization had a truncated U1-SLII capped with a kissing loop motif that has only two cross-strand Watson-Crick GC base pair between two RNA molecules (2KL) [7,30] (Figure 5B). Initially, needle-shaped crystals were grown (only at 4 deg C), which appeared after two hours but dissolved soon after. Examination of the mother liquor revealed that the U1 snRNA was degraded. Further purification yielded more stable crystals, but they did not diffract in-house. Improved crystals were generated by seeding using cat whiskers. Ultimately, the best crystal form of the human U1 snRNP diffracted to 5–6 Å [24]. Initial phases were obtained from MAD phasing with a Ta_6_Br_12_ derivative. The kissing loop interaction was evident in a 5.5 Å experimental map, as RNA is more electron dense (Figure 5C). The crystal belonged to the space group *P*1 with four complexes related by three orthogonal 2-fold symmetry axes (222 symmetry) in the asymmetric unit (Figure 5D). Two kissing loop interactions formed along one of the 2-fold axes. The *222* symmetry also resulted in a helix formed by the complementary base pairing of the 5′ end of the U1 snRNA and its symmetry-related partner, thus mimicking how the 5′ end of U1 snRNA could recognize the 5′ss of the pre-mRNA (Figure 5D). The final structure, built into a multi-domain, multi-crystal averaged 5.5 Å map, revealed the first glimpse of the arrangement of the RNA and protein components of the U1 snRNP [24]. This was achieved by a significant use of anomalous scatters (from seleno-methionine, mercury derivatives, and a single zinc) to build protein into the electron density map [31]. The structure also explained how U1C stabilizes the interaction between a 5′ss and U1 snRNA and how U1-70K facilitates this interaction via its long unstructured N-terminus. The structure was of such high quality that it was possible to use it to guide the engineering of a disulfide cross-link between a 5′ss nucleoside and a proximal cysteine in U1-C [32].

**Figure 6:**
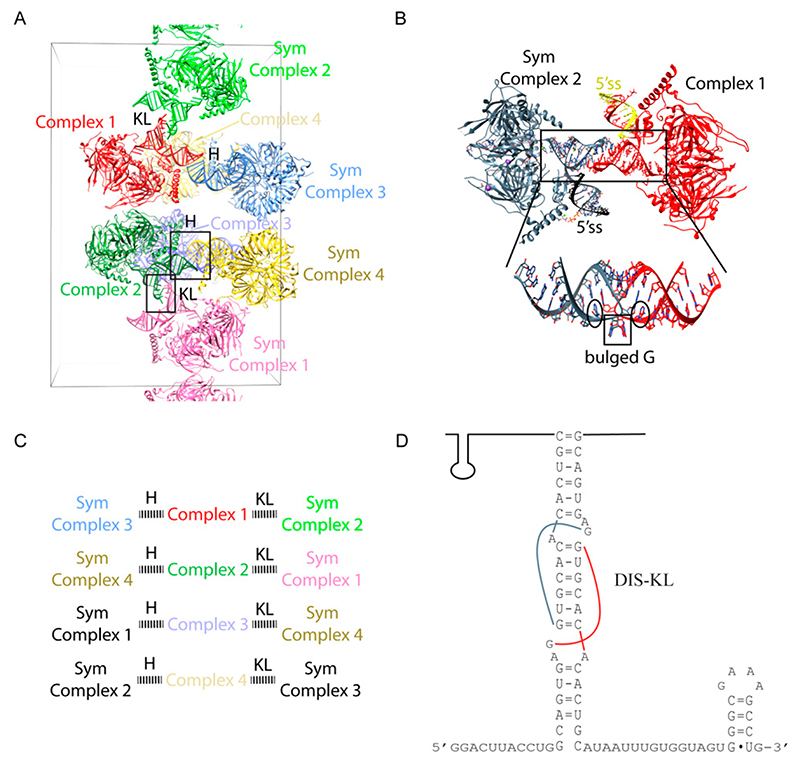
(**A**) The packing arrangement of the minimal U1 snRNP in the *P*2_1_2_1_2_1_ crystal form (PDB:4PJO). Four NCS complexes (Complex 1–4) are present in the asymmetric unit. Complexes 3 and 4 are located behind the page. Only protein-protein interactions are observed between the NCS complexes. Kissing loop interactions (KL) and end-to-end packing of the 5′ss/U1RNA helix (H) are the main crystal packing interactions occurring in this crystal form. Two sets of interactions are depicted in this figure. Complex 1 forms a KL interaction with the symmetry-related Complex 2 and a continuous duplex stacking with the symmetry-related Complex 3. Complex 2 forms a KL interaction with the symmetry-related Complex 1 and a continuous duplex stacking with the symmetry-related Complex 4. An example of this cyclical packing arrangement is summarized in C. (**B**) An example of the KL interaction formed between Complex 1 and the symmetry-related Complex 2. Unlike the original DIS kissing loop complex crystal structure (PDB:1XPE), in which two bulged purines form crystal contacts lateral to the stems, only one purine is bulged out to stack with the equivalent nucleotide. The other purine forms a non-canonical base pair with the unpaired A 3′ to the 6-nt kissing loop complex. (**C**) An example of the packing arrangement involving kissing loops (KL) and continuous end-to-end stacking of the 5′ss/5′U1 duplex (H). Complex 1 interacts with 2–4 in the NCS via protein contacts only. Each NCS complex makes the same packing arrangement with the same set of symmetry-related complexes. The text color for each complex is the same as those depicted in A. Text color in black are complexes that are not shown in A. (**D**) Secondary structure of the RNA construct used in the *P*2_1_2_1_2_1_ crystal form.

Significant effort was taken to improve the diffraction of the crystals by altering the number of base pairs in the SL-II, with no success. In order to obtain better diffracting crystals so as to understand the detailed molecular recognition mechanism of the 5′ ss by U1-C, substantial effort was made to further engineer the U1 snRNA based on the 5.5 Å crystal structure. We first attempted to improve the crystal packing by modifying the 5′-end sequence. We tried changing the length of the 5′-end and adding different palindromic motifs that can self-fold into a stem loop structure capped with a tetraloop or the KL motif (Figure 5E). Subsequently, we tried constructs with native U1-SLII sequence and added back different variants of the U1A protein to the complex, with the hope that the U1A/U1-SLII module will promote more desirable crystal packing [7]. We also attempted to modify U1- SLIII by changing its length and moving the 2KL motif from U1-SLII to U1-SLIII (Figure 5F). In order to design stronger crystal packing, we also tested another kissing loop motif from the dimerization initiation site (DIS) of the HIV-1 genome (DIS-KL) (Figure 5F) [33]. The DIS-KL motif forms a kissing loop complex with more extended base pairs (6 vs. 2 bps); it also has two bulged adenines that can facilitate additional lateral contacts between the stems [7]. While U1 snRNPs reconstituted with these various engineered RNA constructs were crystallizable, we were not able to improve the resolution. The best diffracting crystal with the 2KL motif placed on U1-SLIII with U1A/U1-SLII produced a ~6.6 Å map and showed the scissoring motion of the 4-way junction. The flexibility of the junction and our failed attempts to improve resolution after extensive engineering of the U1 snRNP complex gave us the justification to try more artificial constructs, lacking the 4-helix junction. Eventually, the best diffracting crystals were obtained from a “minimal” U1 snRNP in which the entire 4-helix junction was replaced by one stem loop capped by the DIS-KL motif (Figure 6D). The truncation removed the U1-SLI, the major binding site of the RNA binding domain (RBD) of U170K (residues 100–180), thus drastically reducing the binding affinity of U170K to the particle. Based on backbone tracing using Se-Met anomalous signals of single Se-Met mutants of U170K obtained from the low diffracting crystal form, Pomeranz Krummel et al. modeled the N-terminus of U170K wrapping around the core ring as an unstructured peptide to create a critical interaction with U1C near the 5′ss, consistent with the previous report that showed the N-terminal region of U170K is crucial for U1C assembly [34]. To ensure the incorporation of the U1C protein to the minimal U1 snRNP and reveal the molecular mechanism of how the U1C protein stabilizes the 5′ss binding, the N-terminal 59 residue peptide of U170K was fused to the core protein SmD1. The fusion construct was designed based on the 5.5 Å crystal structure in which the C-terminal end of the unstructured peptide of U170K was mapped nearest to SmD1. This extensive engineering resulted in crystals that diffracted to 3.3 Å [35]. The crystal structure of the minimal U1 snRNP belonged to *P*2_1_2_1_2_1_, with four complexes in the asymmetric unit. Only protein-protein contacts were observed between the NCS complexes. In contrast, RNA-RNA contacts occurred between complexes related by crystallographic symmetry. Each NCS complex formed a repetitive pattern of kissing loop and continuous end-to-end stacking of the 5′ss/U1 duplex with the corresponding crystallographic symmetry-related complex (Figure 6A,C). The DIS-KL interactions occurred at the crystallographic 2 folds (Figure 5C). However, unlike the original crystal structure of the DIS-KL complex in which two purines 5′ to the 6-nt kissing WC pairing flipped out and stacked with a neighboring duplex [7,33], only one of the purines bulged out to stack with the equivalent nucleotide of the kissing complex. The other purine formed a non-canonical base pair with the unpaired A 3′ to the 6-nt kissing WC pairing (Figure 6B). The minimal U1 snRNP crystal structure co-crystallized with a consensus 5′ss oligonucleotide, thus uninfluenced by possible crystal packing of how U1 snRNP recognizes the 5′ss. In addition, the 2.5 Å atomic structure of the remaining U170K (residues 60–216) protein was determined as part of the ternary complex with an RNA fusion construct that had its cognate U1-SLI and the U1A bound U1-SLII, the latter was introduced to promote crystal contacts. U1A participated in crystal packing, interacting with the RRM of U170K. Thus, the detailed atomic architecture of how U1C and U170K stabilize the U1 snRNP/5′ss duplex was finally revealed from these two substructures [35]. The coordinates of both of these U1 snRNPs structures (3CWJ and 4PJO) have been used as the model templates to dock into the pre-B complex of the spliceosome, which reveals the mechanism of how the 5′ss is transferred from U1 snRNP to U6 snRNA in the activated spliceosome [19].

The goal of crystal engineering is to remove structural heterogeneity by deleting flexible regions or introducing crystal contacts to reduce the degree of freedom of certain parts of the molecule. The limitation of the strategy is that the flexibility may have biological significance. In the Nagai group’s decades-long effort toward the structural understanding of the spliceosome, informed decisions were made to engineer various complexes based on available biochemical data at the time. In the case for U1 snRNP, the hairpin U1-SLII/U1A fragment was the first RNA-protein complex determined [12]. However, this substructure was removed to introduce the kissing loop that led to the first successful crystal structure of U1 snRNP [24]. Removing the U1A binding site on the U1-SLII significantly shortened U1-SLII (Figure 5A,B), which would reduce potential structural heterogeneity contributed by multiple orientations of the distal end of the U1-SLII relative to the 4-way junction. Subsequent extensive engineering effort further confirmed that U1-SLII/U1A is inherently flexible as U1-SLII are in different orientations in three available crystal structures that contain this stem-loop (no U1-A at 5.5 Å [24], PDB: 3CW1; with U1-A at 4.4 Å [23], PDB:3PGW; and with U1-A at 6.6 Å [36]). Canonical splicing does not require U1A [28,29], but more recent biochemical data shows that it plays a role in recruiting U1 snRNP in alternative splicing [37,38]. Although the effect of U1-SLII truncation on alternative splicing was not assayed for in our work, the combined structures can rationalize how the flexibility of U1-SLII/U1A is functionally required to accommodate alternative splicing in different cellular conditions. With newer biochemical data, it is conceivable to design a more conformationally homogeneous U1 snRNP in a functional context that necessitates the rigidity of U1-SLII/U1A. For example, the SAM68 protein promotes alternative splicing of the gene *mTor* by recruiting U1 snRNP specifically to intron 5. By binding to its target intronic sequence near the 5′ss and interacting with U1A, SAM68 helps recruit and stabilize U1 snRNP to intron 5 [37]. U1 snRNP in complex with SAM68 and an RNA fragment containing the 5′ss and SAM68 binding site may be a plausible future U1 snRNP design that can result in a higher resolution structure, which can shed light on how the structural plasticity of U1 snRNP enables alternative splicing.

## 5 Future Relevance of Designing Crystal Packing of Spliceosomal Complexes

The design of RNA constructs to promote crystal contacts is still relevant in the splicing field. Despite the Cryo-EM field emerging and developing fast, there is still a need for high resolution crystal structures [39]. The resolution of Cryo-EM structures may not be uniformly high, particularly for peripheral or dynamic components. For example, the activity of helicases plays a major role in remodeling the RNA structures to push the spliceosome in different conformations throughout catalysis. These helicases located in the periphery are poorly resolved in Cryo-EM maps. Another example is the LSm2–8 complex that recognizes the 3′ tail of the U6 snRNA. The LSm proteins are homologous to the Sm proteins and form a heptameric ring that binds RNA. Despite the existence of the number of high resolution Cryo-EM structures of the spliceosome that contain U6 snRNPs, the quality of these Cryo-EM maps for the U6/LSm2–8 complex is insufficient in deducing atomic details. The molecular mechanism of how the Lsm2–8 specifically recognizes the 2′,3′ cyclic phosphate end of the U6 snRNA was not revealed until the recent crystal structures determined at 2.3 Å were published [40].

In the age of Cryo-EM, crystallography can complement Cryo-EM to enhance the completeness and details of the structural information unearthed. Resolution in Cryo-EM depends on the accuracy of alignment (centering and orientation) of the single particles, which is analogous to long-range order in crystals. Better alignment results in better averaging of the boxed images, hence higher resolution of the image reconstruction. The large complexes, by containing more spatial information than the small complexes, are more easily aligned with accuracy. Within large complexes analyzed by Cryo-EM, sub-complexes existing in heterogenous orientations relative to the bulk of well-aligned particle will be blurred in the averaging. Special techniques of image processing such as focused refinement may not be able to recover the lost detail due to local misalignment. As the smaller complexes are more amenable to growth of well-ordered crystals, it may be a general approach to fill in structural details of misaligned sub-complexes by crystallography. Another known barrier to achieving high resolution in Cryo-EM is preferred orientation of the single particles, which causes increasingly incomplete sampling of the Fourier terms with increasing resolution. This is analogous to anisotropic resolution in crystallography. If the preferred orientation cannot be overcome by modifying the surface of the particle, or of the EM grid, or by tilting the grid, etc., then crystallography of the sub-complex should be considered.

Currently, Cryo-EM methodology is still limited in the speed for data collection, thus restricting its practicality for drug screening. Dysregulation in alternative splicing leads to many human diseases [41]. Therefore, discovering molecules that can normalize splicing defects or enhance a weak splice site to compensate for a loss-of-function mutation can be a therapeutic strategy. For example, spinal muscular atrophy is a motor neuron disease due to the deletion of the survival of motor neuron 1 (*SMN1*) gene. The *SMN2* gene is almost identical to *SMN1*, except it is mostly spliced into a non-functional protein by the exclusion of exon 7 [42]. Strategies that promote the inclusion of exon 7 of the *SMN2* pre-mRNA have been explored as one way to treat the disease [42]. Small molecules that bind and stabilize the duplex formed between the 5′ end of the U1 snRNA and the *SMN2* pre-mRNA have been identified as potential drugs. By stabilizing the 5′ss/U1 duplex, the drugs convert exon 7 into a strong splice site [43,44]. Since U1 snRNP plays a key role in splice site selection, U1 snRNP crystals can serve as a drug screening platform for developing compounds that can exert therapeutic effects by manipulating splicing mechanisms.

## Dedication

The authors dedicate this review to their dear friends, collaborators, and mentors—Chris Oubridge and Kiyoshi Nagai. We are indebted to the collaborative environment that Kiyoshi fostered in the laboratory. It has been a great honor to be part of this scientific adventure led by our Sensei (Kiyoshi) and supported by Chris. We have many fond memories of our struggles (for example, spending hours in the cold-room working with U1 snRNP crystals, working around the clock to optimize numerous constructs, getting speeding tickets traveling to the synchrotron), and our eventual successes (first time seeing crystals and RNA backbone density). Their drive, innovation, and perseverance inspired us and contributed significantly to our understanding of the structure-function of RNA-protein interactions and the spliceosome. They are sorely missed and will be remembered.
